# Extranodal Rosai–Dorfman Disease as Isolated Lesion of the Tibia Diagnosed by Fine-Needle Aspiration Cytology: A Case Report

**DOI:** 10.1097/MD.0000000000002038

**Published:** 2015-10-30

**Authors:** Jie Xu, Chun-Hua Liu, Yan-Si Wang, Chang-Xian Chen

**Affiliations:** From the Department of Orthopaedic Surgery, Fujian Provincial Hospital of Fujian Medical University, Fuzhou (JX); Department of Orthopaedic Surgery, Quanzhou Orthopedic-Traumatological Hospital, Fujian University of Traditional Chinese Medicine, Quanzhou (CHL, CXC); and Department of Orthopaedic Surgery, Fuzhou Second Hospital of Xiamen University, Xiamen, Fujian Province, China (YSW).

## Abstract

Few studies have used fine-needle aspiration cytology for the purpose of isolated skeletal Rosai–Dorfman diseases (RDDs) diagnosis.

Herein, we described an extremely rare case of a 56-year-old woman who presented to our hospital with an insidious onset of pain in the right proximal tibia.

The provisional cytologic diagnosis of RDDs was confirmed by a computer tomography-guided core needle biopsy of the lesion. Subsequently, curettage and autogenous iliac crest bone graft were performed successfully. At the 4th year of follow-up her clinical symptoms disappeared, and there was no clinical evidence of lesion recurrence.

Our case highlighted the role of fine-needle aspiration cytology with immunohistochemical studies in the diagnosis of RDDs and the characteristic features of isolated skeletal RDDs in an unusual location, the knowledge of which would help avoid missed or delayed diagnosis in the future.

## INTRODUCTION

Rosai–Dorfman disease (RDD), also called sinus histiocytosis with massive lmphadenopathy, is a non-neoplastic histiocytic proliferative disorder.^1^ It predominantly affects lymph nodes, extranodal manifestation of RDD occurs in ∼25% of cases.^2^ However, isolated skeletal lesions in the absence of lymphadenopathyh, with a total of 14 cases published to date, have been identified; of these, 3 were diagnosed on the basis of cytologic material and the others on the basis of histologic material.^3^ Another case of extranodal RDD as solitary bone lesion is discussed here; we feel that this case is the earliest reported case of RDD that occurred in the proximal tibia and was diagnosed using fine-needle aspiration cytology (FNAC).

## CONSENT

Written informed consent for the images and other clinical information relating to this case report was obtained from the patient.

## CASE HISTORY

**FIGURE 1:**
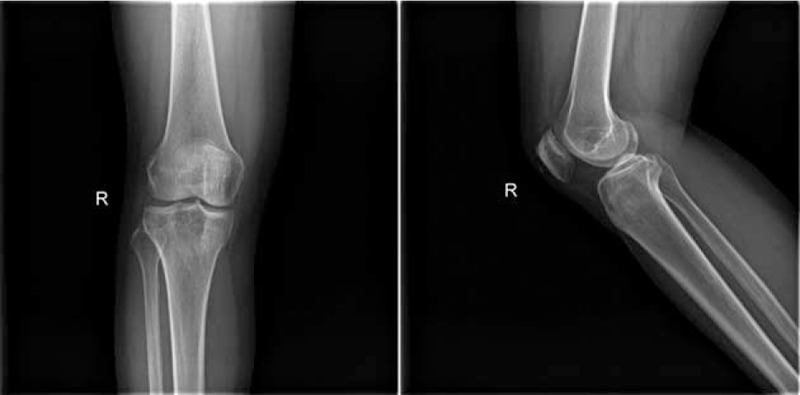
Plain radiograph of the right knee reveals a sharply defined isolated lytic lesion with sclerotic borders. No cortical destruction, periosteal reaction, or soft-tissue component are present.

**FIGURE 2:**
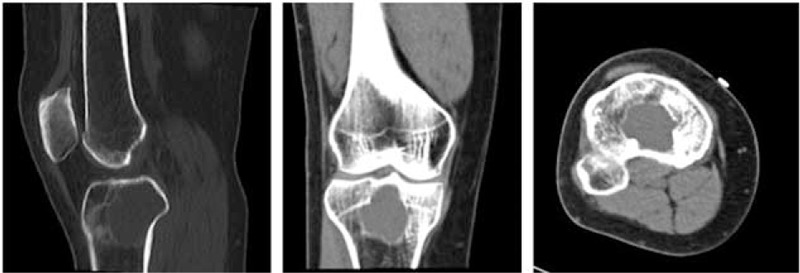
Computed tomography image through the tibial lesion demonstrates lytic lesion within the right proximal tibia has not broken through the cortex, in areas of endosteal erosion, the adjacent cortical bone is sclerotic.

**FIGURE 3:**
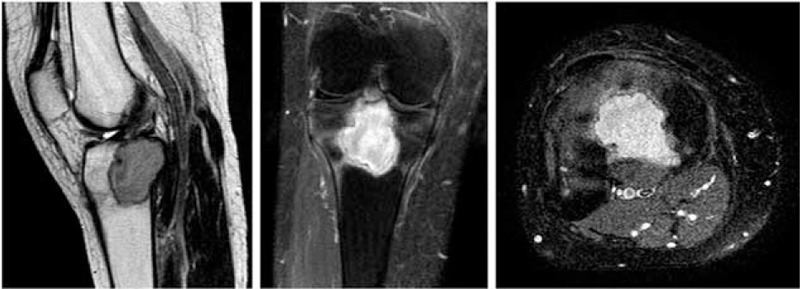
Magnetic resonance images showed the sharply defined lumps and abnormal signal intensity. The lesion is located eccentrically in the posterior aspect of the right proximal tibia and extends close to the articular surface.

A previous healthy 56-year-old woman presented to our hospital with a 1-year history of persistent difficulties in walking, which worsened during 1 month before admission. These difficulties were accompanied by progressive pain in the right proximal tibia which treatment with a nonsteroidal antiinflammatory drug did not improve. She denied trauma, fever, chills, skin rashes, night sweats, and weight loss. Physical examination at the time of admission was remarkable only for an isolated, firm, tender, nonfluctuating, nonerythematous, soft-tissue swelling directly over the right proximal tibia without a noticeable deformity, and peripheral lymphadenopathy. Laboratory examination revealed a moderately elevated erythrocyte sedimentation rate (27 mm/h). Other values, including white blood cell count, C-reactive protein, rheumatoid factor, tumor markers (AFP, CA153, CA199, CA125, CEA), and urinary routine test were within normal ranges. Plain radiograph of the right knee showed a lytic, destructive lesion with sharp border in the proximal tibia (Fig. 1). A computer tomography image through the right tibial lesion revealed the same osteolytic bone lesion (Fig. 2). Subsequently, magnetic resonance images were performed and showed a well-defined intraosseous lesion extending close into the articular surface and involvement of the posterior tibial cortex (Fig. 3). As no additional material was available for further evaluation, a radiological diagnosis of “osteomyelitis, lymphoma, osteogenic sarcoma, or metastatic neoplasms” was rendered.

**FIGURE 4:**
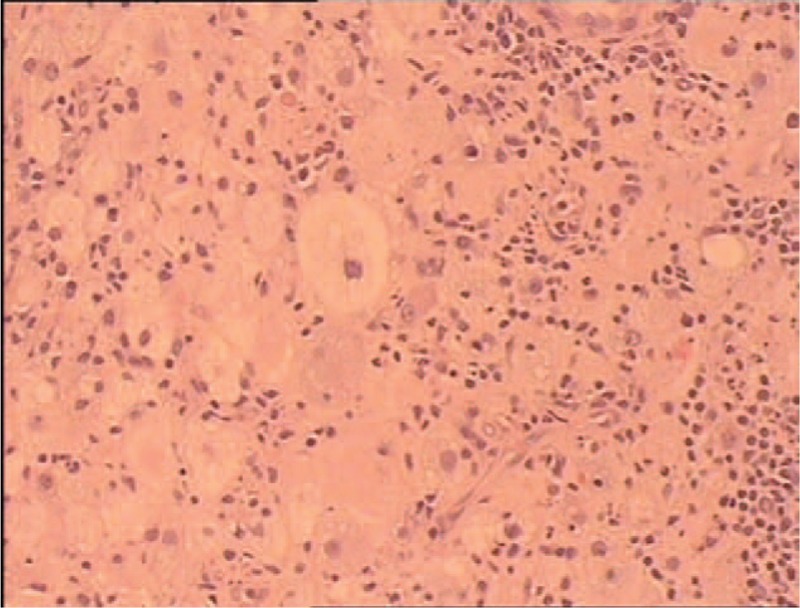
Histopathological picture revealed large histiocytes possessed abundant cytoplasm, rounded nuclei, fine chromatin, and distinct nucleoli (H&E ×200).

Due to the suspicion of neoplastic process, the patient underwent a computer tomography-guided FNAC and a concurrent core needle biopsy of the right proximal tibial lesion in the operating room. Special stain (a modified Papanicolaou technique) on the cytologic material revealed that numerous large histiocytes had been interspersed with different amounts of lymphocytes, neutrophils, and plasma cells. The cytologic features were suggestive of RDD. Subsequently, the biopsy sample was stained with H&E, and immunohistochemical studies were performed. The H&E of the concurrent core needle biopsy revealed the classic features of RDD (Fig. 4); similarly, immunohistiochemical staining showed that the large histiocytes were diffusely positive for S-100 protein, weakly positive for CD68 protein, and remained negative for CD1a protein. Based on these findings, a clinical diagnosis of “extranodal RDD of the tibia” which was reflective of the radiologic or clinical impression was confirmed.

Curettage and autogenous iliac crest bone graft, without radiation therapy and chemotherapy, were advised to perform, since progressive pain in the right proximal tibial lesion and concern for a potential future pathological fracture. Radiographically at the end of 6 months significant reduction in the size of the lesion was noticed and complete resolution of plain X-ray abnormalities when first seen was observed at the end of 1 year, with no evidence of lesion recurrence during 4 years postoperative follow-up.

## DISCUSSION

RDD (sinus histiocytosis with massive lmphadenopathy) was first described in 1969 by Rosai and Dorfman as benign, idiopathic, distinct histiocytic disorder that is comparatively rare and is typified by painless bilateral cervical lymphadenopathy together with pyrexia, leukocytosis, and increase in erythrocyte sedimentation rate.^1^ Currently, the origin of this condition is unclear; however, it is suspected to be caused by a virus.^4^ As per the World Health Organization classification of tumors, these lesions are categorized as a reactive condition of unknown etiology.^5^ Extranodal RDD accompanied by lymphadenopathy is seen in almost half of the cases; however, extranodal manifestation of RDD in the absence of lymphadenopathy is extremely rare.^6^ The skin, respiratory tract, orbital cavity, and the central nervous system are the main extranodal sites involved, followed by the skeleton.^2^ The occurrence of RDD along with isolated skeletal lesions is remarkably rare and is seen in ∼2% of cases, and solitary bone involvement in the absence of lymphadenopathy has been noted in extremely few cases.^7^

The differential diagnosis of extranodal RDD of the bone is occasionally difficult because of the occurrence of clinical signs and symptoms that are nonspecific and because of lesion rarity and the less classic radiologic features observed at times.^3^ To the best of our knowledge, many studies have used FNAC for the purpose of extranodal RDD diagnosis. Although many clinical disorders and diseases of the skeletal system have been described, only 14 cases of isolated skeletal lesions in the absence of lymphadenopathyh have been reported until date; of these, all except 3 by FNAC were diagnosed on the basis of histologic material.^3^ An additional case of extranodal RDD as solitary lesion of the bone is discussed here; we feel that this case is the earliest reported case of RDD that occurred in the proximal tibia and was diagnosed using FNAC.

In the literature that reported the cytologic features of the osseous lesions, classic features of RDD were well described, which was also seen in our case where numerous large histiocytes had been interspersed with different amounts of lymphocytes, neutrophils, and plasma cells.^8^ Characteristically, those histiocytes contained abundant cytoplasm and distinct nucleoli with fine chromatin, and demonstrated conspicuous emperipolesis of lymphocytes, plasma cells, and nuetrophils.^6,9^ Immunohistochemically, the large histiocytes were intensely positive for S-100 protein, weakly positive for CD68 protein, and remained negative for CD1a protein, which confirmed RDD diagnosis.

Although the lesion usually undergoes spontaneous resolution, most cases of RDD typically follow a waxing and waning of the clinical course.^9^ A variety of ways have been used and described for the treatment of RDD involvement of the bone, including corticosteroids, chemotherapy, radiotherapy, surgical curettage, or resection; recommended therapeutic modality, if possible, contains the clinical observation.^10^ As in our case, in the rare case of extranodal site presenting with progressive pain and the difficulty in walking, surgical curettage and autogenous iliac crest bone graft may be necessary. Importantly, surgical intervention seems to be the preferable treatment option for symptomatic RDD, as most cases of isolated skeletal RDD in previously reported studies healed completely after the totally surgical curettage or resection.^10^ Simply put, skeletal involvement of RDD is seldom fatal, and the long-term prognosis is good for isolated extranodal lesion.^5,6^

## CONCLUSIONS

To our knowledge, there have been no reports describing isolated skeletal RDD that occurred in the proximal tibia and was diagnosed using FNAC. We demonstrate the characteristic features of isolated skeletal RDD in an unusual location and recommend that FNAC with immunohistochemical studies can be a good option in the diagnosis of extranodal RDD of the bone, the knowledge of which would help avoid delayed or missed diagnosis in the future.
